# Olfaction and Anxiety Are Differently Associated in Men and Women in Cognitive Physiological and Pathological Aging

**DOI:** 10.3390/jcm12062338

**Published:** 2023-03-17

**Authors:** Filippo Cieri, Nicoletta Cera, Aaron Ritter, Dietmar Cordes, Jessica Zoe Kirkland Caldwell

**Affiliations:** 1 Department of Neurology, Cleveland Clinic Lou Ruvo Center for Brain Health, Las Vegas, NV 89106, USA; 2 Laboratory of Neuropsychophysiology, Faculty of Psychology and Education Sciences, University of Porto, 4200-135 Porto, Portugal; 3 CIBIT—Coimbra Institute for Biomedical Imaging and Translational Research, University of Coimbra, 3000-548 Coimbra, Portugal; 4 Department of Brain Health, University of Nevada, Las Vegas, NV 89154, USA; 5 Department of Psychology and Neuroscience, University of Colorado, Boulder, CO 80309, USA

**Keywords:** olfaction, anxiety, sex difference, aging, Parkinson’s disease

## Abstract

Background: Olfaction impairment in aging is associated with increased anxiety. We explored this association in cognitively healthy controls (HCs), Mild Cognitive Impairment (MCI) and Parkinson’s disease (PD) patients. Both olfaction and anxiety have sex differences, therefore we also investigated these variances. Objectives: Investigate the association of olfaction with anxiety in three distinct clinical categories of aging, exploring the potential role of sex. Methods: 117 subjects (29 HCs, 43 MCI, and 45 PD patients) were assessed for olfaction and anxiety. We used regression models to determine whether B-SIT predicted anxiety and whether sex impacted that relationship. Results: Lower olfaction was related to greater anxiety traits in all groups (HCs: *p* = 0.015; MCI: *p* = 0.001 and PD: *p* = 0.038), significantly differed by sex. In fact, in HCs, for every unit increase in B-SIT, anxiety traits decreased by 7.63 in men (*p* = 0.009) and 1.5 in women (*p* = 0.225). In MCI patients for every unit increase in B-SIT, anxiety traits decreased by 1.19 in men (*p* = 0.048) and 3.03 in women (*p* = 0.0036). Finally, in PD patients for every unit increase in B-SIT, anxiety traits decreased by 1.73 in men (*p* = 0.004) and 0.41 in women (*p* = 0.3632). Discussion: Olfaction and anxiety are correlated in all three distinct diagnostic categories, but differently in men and women.

## 1. Introduction

Olfaction has an important difference compared to the other sensory systems. Unlike the other senses, olfactory neurons project directly to the limbic system, bypassing the thalamus. This feature may explain strong associations between olfaction and emotion [1,2]. Since emotions and moods, such as anxiety, play a key role in cognitive aging, this uniqueness of olfaction can play a critical role in the screening and diagnosis of cognitively pathological aging, in a society where the rising age of the general population leads to suffering for patients and family members, with a relative economic and social cost.

Along with other sensory systems, olfaction is impaired in aging, a condition known as hyposmia, which occurs in over half of individuals between the ages of 65 and 80 and between 62–80% of those over the age of 80 [3] Since hyposmia is a typical symptom of Parkinson’s disease (PD), affecting 75% to 90% of patients, olfactory tests represent a sensitive screening for this disorder and have been incorporated into the established International Parkinson and Movement Disorder Society criteria for PD and prodromal PD [4]. However, olfactory screening is still little used, unlike screening for anxiety, which is extensively used in the exploration of cognitive and behavioral functions in the older individual, due to its important implications in the individual life.

The relationship between emotion/mood and olfaction is well-established [5], sharing similar evolutionary history and brain anatomical structures, particularly in the limbic regions [6]. Olfaction perception also has been demonstrated to influence mood [7] and stress response [8]. Studies on the association between olfaction and anxiety have been conducted on obsessive-compulsive, panic disorder and post-traumatic stress disorder (PTSD) patients (see [9] for a review) based on structural overlap in brain regions involved in olfaction and anxiety, like the orbitofrontal cortex, amygdala, hippocampus, and insula [10].

Coronavirus disease 2019 (COVID-19) has worldwide re-emphasized the association between olfaction and psychiatric symptoms, particularly anxiety. In fact, in the severe acute respiratory syndrome coronavirus 2 (SARS-CoV-2), patients are affected by decreased sense of smell and taste [11] in conjunction with psychiatric symptoms, especially anxiety [12]. 

Aging, olfactory functions, and anxiety have different impacts based on sex. Sex differences in aging are well known, especially in Alzheimer’s disease (AD; [13,14,15,16,17], where women are 2/3 of the patients, and in PD, where both incidence and prevalence of PD are 1.5–2 times higher in men than in women [18]. In humans, there is an increasing sex ratio associated with aging such that there are ~50 men per 100 women among 90-year-olds [19] because men physiologically age faster than women. Olfactory functions seem to follow a similar pattern, declining more rapidly in men [20]. Women typically outperform men on olfactory tasks during normal aging, but it is not clear whether this is due to differences in peripheral sensory function or central cognitive processing of olfactory information [21]. These results suggest that age-related functional changes in men and women may follow a different path and time course, but this hypothesis has not been explicitly investigated in cognitively physiological and pathological aging. Moreover, although the association between olfaction and anxiety is well known, as well as sex differences in aging, these specific factors have not been studied in clinical and preclinical populations of older individuals.

The aim of the present study was three-fold. First, we wanted to show that olfactory function and anxiety (state and traits) are negatively correlated. Secondly, we wanted to show that this anticorrelation is present in HCs, MCI and PD, as three different diagnostic categories of aging. Finally, we wanted to explore sex differences, assuming that the anticorrelation between olfaction and anxiety follows different courses in men and women of aging populations.

## 2. Materials and Methods

Participants: This study was authorized by the Cleveland Clinic Institutional Review Board, Study #15-888, The Relationship between Neuropsychological Testing and MRI, PET and Blood Biomarkers in Neurodegenerative Disease (NIH), following the principles outlined in the Declaration of Helsinki (https://www.wma.net/policies-post/wma-declaration-of-helsinki-ethical-principles-for-medical-research-involving-human-subjects/, accessed 10 January 2023). We included 117 subjects from the Center of Biomedical Research Excellence (COBRE), with 29 HCs (62.1% women = age mean 71 ± 7.0 SD; men = 70.6 ± 6.8 SD), 43 MCI patients (34.9% women = age mean 73.0 ±5.3 SD; men 74.3 ± 5.9 SD) and 45 PD-MCI patients (40% women = age mean 69.44 ± 7.04 SD; men age mean 71.56 ± 7.47 SD). Our inclusion criteria for HCs, MCI and PD are as follows:

HCs: (1) Age between 55–90; (2) visual and auditory acuity; (3) good general health; (4) score of 12 or greater on the Montreal Cognitive Assessment (to ensure adequate ability to complete neuropsychological testing); (5) no signs of MCI, PD, or other dementia.

MCI: (1) Subjective memory complaints reported by themselves, study partner, or clinician; (2) objective memory loss defined as scoring below an education-adjusted cut-off score on delayed recall of Story A of the WMS-R Logical Memory Test; (3) global CDR score of 0.5; (4) general cognitive and functional performance sufficiently preserved such that a diagnosis of dementia could not be made at the time of screening.

PD: Parkinsonism is defined as bradykinesia, in combination with either rest tremor, rigidity, or both, considering supportive and absolute exclusion criteria [4]. The MDS-Unified Parkinson’s Disease Rating Scale (UPDRS) was used, with no autopsies for the diagnosis confirmation.

All the subjects were screened for psychiatric and neurological conditions at the Cleveland Clinic Lou Ruvo Center for Brain Health, the assessment, including the neuropsychological and the olfaction tests, took place at the same visit. All participants provided written informed consent.

Olfaction: We used the Brief Smell Identification Test (B-SIT 3rd edition; [22]), the most widely used olfactory test, normed and accurate, which provides an indication of smell loss associated with disorders, such as those involving memory loss and other sensory issues. It is composed of twelve “scratch and sniff” odorants, with a score range of 0–12, where higher scores represent greater olfactory function.

Anxiety: We used the State Traits Anxiety Inventory (STAI), a commonly used test allowing measuring of traits and state anxiety [23]. It is used in clinical settings to assess anxiety and to distinguish it from depressive syndromes.

Statistical analysis: Statistical analyses were conducted with IBM SPSS version 23. In HCs, MCI, and PD groups, differences between men and women were assessed using one-way ANOVAs for continuous variables (ages and education). The categorical variable “sex” (men or women) has been compared using the Yates corrected Chi-square test. We used a regression model for B-SIT predicting STAI traits and state, and an interaction regression model to determine the interaction between sex and olfaction predicting anxiety traits in all three groups.

To assess the differences among the age, education, B-SIT and STAI trait and state scores for gender separately, two one-way MANOVA were calculated. Moreover, a Duncan’s post hoc test on the univariate offered significant results.

## 3. Results

**Figure 1:**
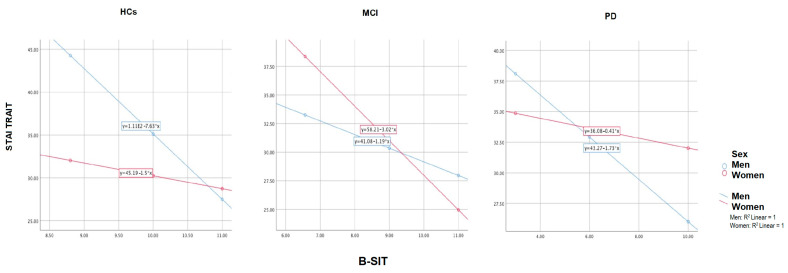
HC, MCI, PD partial regression plot of B-SIT*Sex interaction predicting STAI Traits (controlled for age and education).

We found no significant age difference between HCs and PD subjects, but we found a significant difference between these two groups and the older MCI group (F_(2)_ = 3.463; *p* = 0.035). We found no education difference between groups (F_(2)_ = 2.749; *p* = 0.068). No significant sex differences were observed (Table 1). Loss of olfaction has a significant negative correlation with anxiety state only in MCI: *r* _(38)_ = −0.39, *p* = 0.006, neither in HCs= *r* (27) = −0.13, *p* = 0.238; nor PD= *r* (43) = −0.11, *p* = 0.226. On the other hand, olfactory function was significantly anticorrelated with anxiety traits in all the groups. Table 1 shows our regression model for B-SIT predicting STAI traits. This model shows a significant result in HCs, with β= −2.96 (−5.29, −0.63) *p* = 0.015; MCI, β= −1.93 (−3.05, −0.82) *p* = 0.001 and PD, β= −1.06 (−2.05, −0.06) *p* = 0.038 (adjusted for age, sex, and education). These findings differed by sex. In our three different groups, we found that in HCs (Figure 1), for every unit increase in B-SIT, anxiety traits decreased by 7.63 in men (*p* = 0.009) and 1.5 in women (*p* = 0.225). Conversely, in MCI (Figure 1), for every unit increase in B-SIT, anxiety traits decreased by 1.19 in men (*p* = 0.048) and 3.03 in women (*p* = 0.0036); in PD, for every unit increase in B-SIT, anxiety traits decreased by 1.73 in men (*p* = 0.004) and 0.41 in women (*p* = 0.3632).

**Figure 2:**
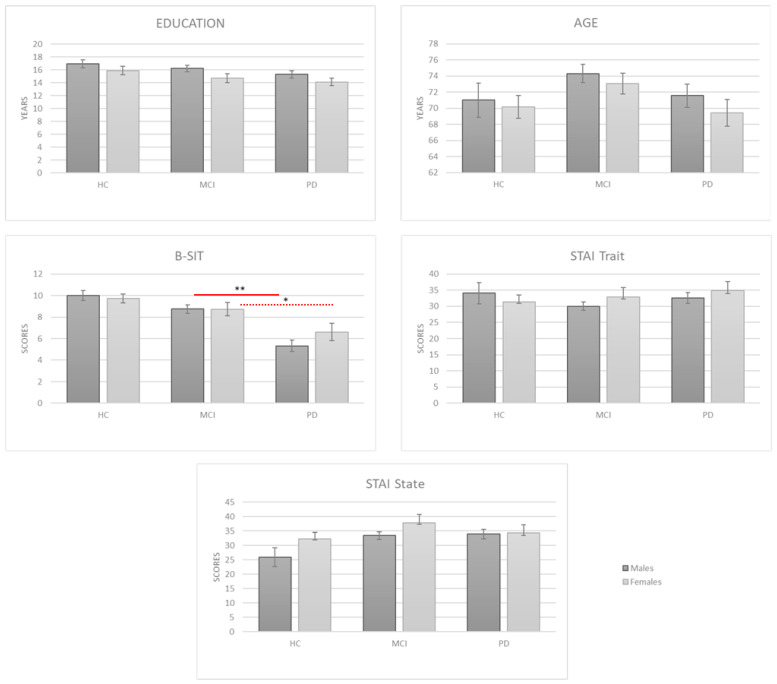
Error bars denote the standard errors and the * = *p* > 0.05 and ** = *p* < 0.001.

Moreover, no significant differences have been found in the age and education of the two sexes. Interestingly, men (*p* = 0.0001) and women (*p* = 0.004) showed significant differences in the B-SIT scores (Table 1). A post hoc test revealed significantly lower scores for PD than MCI and HCs. Particularly, in the men group this difference between PD and MCI was stronger (*p* = 0.0001) than in the women group (*p* = 0.035—Figure 2). Moreover, the MCI group has significantly lower score for the memory delay recall (RAVLT Delay Recall *p* < 0.05).

In HCs for every unit increase in B-SIT, STAI Traits decrease by 7.63 in men and 1.5 in women. In MCI for every unit increase in B-SIT, STAI Traits decrease by 3.03 in women and 1.19 in men. In PD for every unit increase in B-SIT STAI Traits decrease by 0.41 in women and 1.73 in men.

## 4. Discussion

Among all the sensory systems, olfaction is considered the most phylogenetically ancient, with direct anatomical connections with the limbic system, particularly the hippocampus [5,6]. This olfactory-limbic structural connection is evident in the overlap between the limbic structures involved in olfaction and emotional processing [24,25] to the point that olfactory stimulation has been used as a method for mood induction [26].

Due to the partial neural overlap of olfaction and anxiety, and research evidence of both decline of olfaction in anxiety disorders and increased anxiety in hyposmic patients, we hypothesized a negative correlation between olfaction and anxiety, regardless of cognitive status (HCs, MCI, and PD). We further assumed that this association would show a specific sex difference. The present study largely confirmed these hypotheses.

Firstly, we have shown that olfactory impairment is negatively correlated with anxiety traits. The reason why olfaction has an anticorrelation with anxiety traits in all our three groups, and not with the anxiety state, may be due to the fact that while the trait is more stable—a feature shared with olfaction—the state is temporally restricted and may fluctuate significantly with environmental rather than intrinsic influence.

Secondly, we have observed that this negative correlation between olfaction and anxiety traits is present in all three distinct diagnostic groups, confirming our second hypothesis. In other words, more anxiety traits are correlated with less olfactory functions, in physiological (HCs) and non-physiological (MCI and PD) neurocognitive aging.

Moreover, since several studies have shown differences between men and women in olfactory functions [21,26,27], we explored sex differences in the association between olfaction and anxiety, finding that anxiety traits are anticorrelated with olfaction, in both sexes but to different extents in men and women. In the HCs, better olfaction was correlated with fewer anxiety traits significantly more in men compared with women. A similar pattern was observed in the PD group, with the strongest anticorrelation of olfaction-anxiety traits in men compared with women. Conversely, in MCI subjects, although both sexes have shown an anticorrelation between olfaction and anxiety traits, we noticed an opposite trend by sex compared to HCs and PD, with the highest anticorrelation in women compared with men. One of the reasons for this divergence might be due to the fact that MCI is a broad and general category, a more heterogeneous disease, composed of different entities. Accordingly, with this premise, among MCI individuals there might be subjects who convert to Alzheimer’s Disease (AD), as the most widespread form of dementia, affecting women in almost 70% of cases [28]. Therefore, women may have an initial pathophysiological manifestation of AD, with a relatively greater association between impaired sense of smell and anxiety, compared with men. Furthermore, women may be receiving a diagnosis at later stages of pathology due a better verbal memory, a form of cognitive reserve [14,16]. As we mentioned, even though several studies have demonstrated different behavioral results between men and women in olfactory functions [21,26,27], it is not clear whether this is due to differences in peripheral sensory function or central cognitive processing of olfactory information, and this aspect deserves more investigation.

Another important aspect of this difference between peripheral sensory and central cognitive processing is related to our three distinct groups (HCs, MCI, PD). In fact, we need to be cautious in our interpretations since the MCI group has a memory deficit and this factor can impact the olfactory identification in this group. On the other hand, our PD patients do not have memory impairments, showing typical PD olfaction impairment.

The BSIT score in literature is associated with a higher level of AD pathology on autopsy (measured by a composite measure of cortical amyloid plaques and NFTs), after controlling for age, sex, education, time from olfactory testing to death, APOE e4 carrier status and episodic memory. This finding supports the notion that odor identification ability is linked with the pathologic manifestations of AD, even in asymptomatic individuals [29]. In our study, we wanted to investigate the association between the loss of olfaction and anxiety measures, exploring potential differences between men and women. In other words, we were interested in the demonstration that regardless of the presence of clinical manifestation of pathology (HCs, MCI, PD) olfaction was associated with anxiety and this association might be different between women and men, as we have shown.

It is established that olfaction can affect mood and stress response [8], and as we mentioned, it is also known that anxiety disorders, such as PTSD, are associated with olfaction impairment. Authors who have used functional Magnetic Resonance Imaging (fMRI) have shown an aging effect in which activation in olfactory-related structures, decreases in older adults compared to young healthy subjects [30]. In middle-aged subjects, a more recent fMRI study showed significant age- and sex-related decline in second-order olfactory structures, with men displaying significant aging effects [31], consistent with our results, at least in our more diagnostic-specific groups (HCs and PD). Significant aging and sex effects have been also found by another recent fMRI study [32] in which both sexes are affected by olfaction impairment associated with aging, but with women manifesting a different mechanism to compensate for age-related olfactory activation decline.

These sex-olfactory differences are consistent with our results, and with evidence from PD literature. In fact, male PD patients report more sadness, mood issues, loss of interest, taste/smelling difficulties, and anxiety, compared to healthy control men [33]. The prevalence of anxiety in this diagnosis is 31%, much higher than reported in the general population or other medically ill patients [34]. Additionally, individual variations in the olfactory performance of PD patients have been associated with sex [35]. Specifically, men with PD have greater and more frequent taste/smelling problems compared to women affected by the same pathology [33].

Smell impairment has a not negligible impact on quality of life. In the general population, hyposmic older adults are more likely to experience depressive symptoms, due to impairment of food and drink enjoyment and socializing [36], but the effect of anxiety is less clear and explored. Our results provide support for an association between sensorial decline and a general neuropsychological state [37] which deserves further investigation.

Olfactory assessment is a promising tool to study the central nervous system. Our approach can be translated into clinical applications and assessments, with the aim to find novel treatments and therapies. The negative correlation between olfaction and anxiety may have an important role in the investigation of neurological and neuropsychiatric disorders, anticipating the diagnosis of possible neurodegenerative disorders. The role of sex in this association can help elucidate pathophysiological mechanisms and guide the development of new therapeutic modalities.

Our study has limitations. The sample size is small. Also, we do not have amyloid data for all our subjects, which limits our investigation and explanation power. Moreover, diagnostic certainty in PD is impossible during life; between 75% and 95% of patients diagnosed with PD by experts have their diagnosis confirmed on autopsy [4]. Finally, this hypothesis needs longitudinal data to investigate the actual decline of the olfactory functions.

Nevertheless, our work has also strengths. There is a paucity of studies that explore sensory and emotional/feelings/mood factors associated with olfaction and similar scarcity of explicit sex difference investigation in aging. To the authors’ best knowledge this is the first study that explored the association between olfaction and anxiety in cognitively physiological and pathological aging, pointing out the role of sex in this correlation.

Future studies aimed at elucidating the interplay between neurodegenerative changes and pathologic accumulation of amyloid may account for the fact that neurodegeneration is more closely associated with worse cognition than amyloidosis [29,38,39,40]. In this perspective, the role of olfaction is important to understand whether its impairment is closer to a worse cognition or amyloid accumulation. Olfactory assessment can be a promising, valuable, and economical research, as well as a clinical tool in the study of the central nervous system, exploring correlations between sensory impairment and general neuropsychological health, helping elucidate pathophysiological mechanisms of neurological and psychiatric disorders, guiding the development of new potential therapy.

## Figures and Tables

**Table 1:** Descriptive and regression analysis for the variables STAI Traits and B-SIT, adjusted for age, gender, and education. *p*-values for the adjusted models, adjusted for age, gender, and education.

	HC				MCI				PD			
	M		W		M		W		M		W	
N (%)	11	(37.93)	18	(62.06)	28	(65.11)	15	(34.88)	27	(60)	18	(40)
Age in years, Mean ± S.D	71.00	±7.03	70.17	±5.97	74.32	±5.98	73.07	±5.01	71.56	±7.47	69.44	±7.04
Education in years, Mean ± S.D	16.91	±2.02	15.89	±2.83	16.21	±2.50	14.73	±2.74	15.30	±2.98	14.11	±2.42
B-SIT Score Mean ± S.D	10.00	±1.55	9.72	±1.81	8.75	±2.08	8.73	±2.40	5.33	±2.80	6.61	±3.45
STAI Trait Score Mean ± S.D	34.09	±10.89	31.28	±9.28	30.04	±6.82	32.92	±10.63	32.59	±8.50	34.83	±12.00
STAI State Score Mean ± S.D	25.82	±7.08	32.33	±11.89	33.44	±12.73	37.85	±11.85	33.93	±9.39	34.33	±12.17
Regression analysis for the variables “STAI and B-SIT”												
Group	Unadjusted				Adjusted				*p* Value			
HC	−2.26 (−4.37, −0.15)				−2.96 (−5.29, −0.63)				**0.015**			
MCI	−1.64 (−2.74, −0.54)				−1.93 (−3.05, −0.82)				**0.001**			
PD	−0.57 (−1.59, 0.44)				−1.06 (−2.05, −0.06)				**0.038**			
Yates corrected χ^2^ to assess sex differences												
	χ^2^				df				*p* Values			
HC	0.28				(1)				0.274			
MCI	1.19				(1)				0.595			
PD	0.41				(1)				0.524			
One-way Multivariate Analysis of Variance for age and education for the sex												
	Wilks Lambda (λ)				F		df		*p* Values			
Males	0.906				1.55		(4.124)		0.191			
Females	0.864				1.77		(4.94)		0.140			
One-way Multivariate Analysis of Variance for age and education for B-SIT and STAI												
	Wilks Lambda (λ)				F		df		*p* Values			
Men	0.485				8.71		(6.120)		**0.0001**			
B-SIT follow up ANOVA					20.62		(2.62)		**0.0001**			
STAI trait follow up ANOVA					1.14		(2.62)		0.32			
STAI state follow up ANOVA					2.51		(2.62)		0.08			
Women	0.725				2.55		(6.88)		**0.024**			
B-SIT follow up ANOVA					6.09		(2.46)		**0.004**			
STAI trait follow up ANOVA					0.80		(2.46)		0.454			
STAI state follow up ANOVA					0.49		(2.46)		0.611			

Significant *p* values are marked in bold. Abbreviations: MCI = Mild Cognitive Impairment; HCs = Healthy Controls; PD = Parkinson’s disease; SD = Standard Deviation.

## Data Availability

Raw data were generated at the Cleveland Clinic, Lou Ruvo Center for Brain Health. Derived data supporting the findings of this study are available from the corresponding author [F.C.] on request.
